# Risk of mental health conditions in bereavement: a population-based analysis of lung cancer spouses

**DOI:** 10.3389/fpubh.2025.1539180

**Published:** 2025-05-12

**Authors:** Djin L. Tay, Kline Dubose, Jonathan Chipman, Lee Ellington, Malek Alnajar, Eli Iacob, Caroline Stephens, Katherine A. Ornstein

**Affiliations:** ^1^College of Nursing, University of Utah, Salt Lake City, UT, United States; ^2^Department of Population Health Sciences, School of Medicine, University of Utah, Salt Lake City, UT, United States; ^3^School of Nursing, Johns Hopkins University, Baltimore, MD, United States

**Keywords:** bereavement, neoplasms, caregiver stress, mental health, epidemiologic studies

## Abstract

**Background:**

Caregiving to lung cancer patients is distressing, isolating, and associated with a high burden of anxiety and depression. However, few population-based studies in the U.S. have examined the risk of mental health conditions (MHCs) among spouses of lung cancer patients after the death of their partner. Guided by Anderson’s Behavioral Health Utilization model, we examined the role of sex, pre-bereavement MHC, and decedents’ healthcare utilization on the risk of having a diagnosed MHC after the death of a lung cancer patient.

**Methods:**

This retrospective cohort study linked state-wide health facility records of 1,224 dyads—deceased lung cancer patients and their bereaved spouses (824 female, 400 male)—in Utah between 2013 and 2021. Bereavement-related mood/stress-related conditions were identified for spouses using diagnostic codes (starting from day 1 following the patients’ deaths). The Kaplan–Meier curves and Cox proportional hazard models were used to estimate the risk for a composite outcome of MHC/death and the risk of MHC, after adjusting for censorship due to death and controlling for covariates.

**Results:**

The majority of spouses were aged 65+ (female: 67%; male: 33%), white/non-Hispanic (female: 89%; male: 90%), and urban-dwelling (female: 69%; male: 71%). Spouses experienced 374 events (MHCs/death) across the follow-up period. Adjusting for census-tract level income, cancer stage, insurance, censoring due to death, and the interaction between sex and MHC, spouses with preexisting MHCs had 4.09 times higher risk of developing MHCs during bereavement (95% CI: 2.70, 6.19) compared to spouses without pre-existing MHCs. Spouses of decedents with some college education (aHR: 0.68, 95% CI = 0.48–0.97) and longer survival (aHR: 0.85, 95% CI = 0.74–0.99) had a lower risk of MHCs compared to those of decedents with high school education and shorter survival.

**Discussion:**

This population-based study supports evidence for multi-level risk factors associated with having MHC after the death of a spouse with lung cancer. Findings suggest the need for targeted bereavement support for subgroups of spouses at greater risk of MHCs.

## Introduction

Cancer caregivers, particularly those supporting patients at the end of life, are a vulnerable population with increased mental health risks (1, 2). Research shows that partners of cancer patients experience anxiety and depression at levels higher than the general population and comparable to those of the cancer patients themselves (3–5). In addition to poorer self-reported mental health, cancer caregivers experience greater psychiatric morbidity before and after bereavement. Population-level studies from other countries have established that spouses of cancer patients have a 30% higher risk than spouses of non-cancer patients of developing new-onset substance abuse, depression, or stress-related conditions within the year following a cancer diagnosis. A 29% increased risk of these mental health diagnoses in bereavement persists, even after adjusting for confounders and other pre-existing mental health diagnoses (6). Similarly, in the United States (US), analyses of commercial insurance claims data found that, compared to non-cancer caregiver controls, almost 2 in 10 (19.6%) cancer caregivers had new mental health diagnoses in the year after losing their partner (7).

Lung cancer is characterized by a high rate of recurrence, low survival (8), and distressing symptoms that contribute to poor quality of life for patients and family caregivers (9–11). The intense symptom management and stigma associated with lung cancer may be particularly isolating for spouse caregivers (11, 12). Lung cancer patients have a higher prevalence of anti-cancer treatment utilization at the end of life (13), which has been associated with poorer mental health among cancer caregivers (14, 15). Analyses of cancer caregivers covered under the same health insurance policy found that caregivers of lung cancer patients were more likely to be diagnosed with a new mental health disorder following the patient’s diagnosis compared to caregivers of non-lung cancer patients (18.9% vs. 10%) (7).

During cancer caregiving, wives have been observed to report poorer mental health than husbands (16). However, studies report mixed findings by gender in bereavement. While the loss of a spouse is among the most distressing life events across genders, husbands experience greater overall physical health impacts compared with wives after losing their spouse (17–19). Yet, with regard to mental health, the findings are less clear. While wives have been reported to experience a prolonged trajectory of distressing symptoms compared with husbands in population studies in Denmark (20), the opposite finding has been observed in a Korean cohort (21). Additionally, symptoms may also differ by gender and time. In the acute bereavement phase, husbands exhibit greater initial shock, while wives exhibit greater long-term psychological resilience around the 1-year mark (22). However, these mixed findings regarding gender could be due to the greater participation of female individuals in caregiving research, as well as the influence of social norms that facilitate female caregivers’ expression of distress and mental health care (23), highlighting the need to examine gender effects in bereavement using population-based approaches.

**Figure 1:**
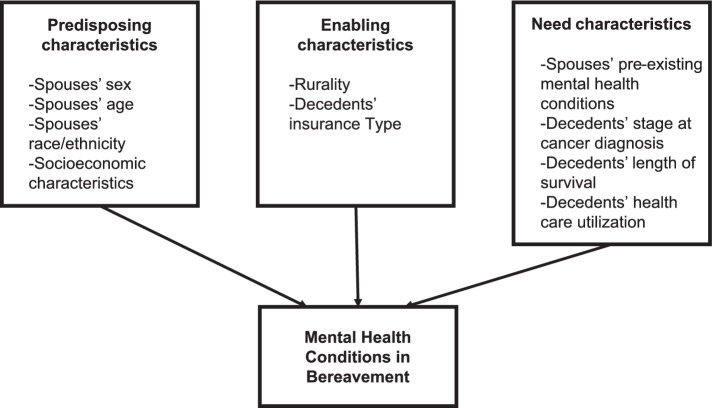
Adapted Anderson Behavioral Health Model for spouses’ bereavement mental health utilization.

While the majority of population-based studies on bereavement have been conducted outside of the US (6, 24), findings from these studies may be less generalizable to the US healthcare system. Examining the risk factors associated with MHCs among lung cancer spouses, a population vulnerable to the mental health impact of caregiving is needed to provide scientific evidence for targeted bereavement interventions in this population. Given these gender-based differences in bereavement mental health, we sought to focus on the relationship between gender and mood- and stress-related mental health diagnoses after the loss of a partner to cancer. Guided by Anderson’s Behavioral Health Model (25), we tested the hypothesis that predisposing characteristics (female sex) and need characteristics (spouses’ pre-existing mental health conditions and decedents’ healthcare utilization) would be associated with the risk of bereavement MHCs (Figure 1).

## Methods

This secondary analysis used data from bereaved spouses and partners of decedents diagnosed with lung cancer between 2013 and 2018 in Utah identified with the Immunotherapy, Palliative, End of Life Treatment Utilization, and Spousal Outcomes (ImmPETUS) cohort (23). ImmPETUS was developed to facilitate the study of changes in cancer patients’ end-of-life and primary caregiver health utilization during a period of rapid adoption of immunotherapies in advanced cancers. The ImmPETUS cohort is a linked dataset comprising demographic, clinical, end-of-life, and spousal caregiver data that combine several key population datasets in Utah: the Utah Population Database’s (UPDB) statewide health facility data from the Utah Department of Health, the Utah All-Payer Claims Database (APCD), and the Utah Cancer Registry (UCR).

### Data sources

#### UPDB

The UPDB is a statewide database that enables the linkage of health and family records from statewide health registries, sociodemographic data, vital records, and genealogical data for all Utah residents at the individual level. The UPDB health facility data are complete as they contain all inpatient, ambulatory surgery, and emergency department administrative data for every encounter in the state, regardless of insurance coverage, including encounters among the uninsured population. However, the UPDB’s health facility data does not capture administrative data from care settings outside of inpatient, ambulatory surgery, and emergency department settings, such as physician visits.

#### APCD

The Utah APCD contains all administrative data, including pharmacy records for the insured population, Medicaid, and commercial Medicare Supplemental and Advantage plans for older adults aged 65 years and above. The APCD does not collect data from non-commercial Medicare plans, health plans covering fewer than 2,500 individuals (26), the Veterans Health Administration or TRICARE system (serving active service members and veterans), Indian Health Services (tribal and urban Indian health programs), and individuals who self-pay are underrepresented (27). As such, the data collected by the APCD represent approximately 60%–70% of Utah’s non-Medicare population (28).

#### UCR

The UCR collects, stores, and manages cancer surveillance data in Utah for the Surveillance, Epidemiology, and End Results (SEER) program of the National Cancer Institute. The UCR captures data on cancer staging, diagnosis, and sociodemographic data current at the time of the index cancer diagnosis. According to the SEER program, cancer data from the UCR are complete and valid (29).

To maximize coverage of the population and data completeness, we linked the UPDB health facility, Utah APCD, and UCR data for the sample. Eligible cancer patients (1) had a record in the UCR; (2) had a diagnosis of lung or bronchus cancer between 2013 and 2018; and (3) were at least 18 years of age or older at the time of cancer diagnosis. Spouses and partners aged 18 and above were identified with UPDB’s family groups, which are derived using a combination of marital records and children’s birth records. Cancer patients with a non-natural cause of death (e.g., an accident) were excluded.

### Measures

The variables were selected *a priori* and informed by Anderson’s Behavioral Health Utilization Model (25). We examined the associations between *predisposing* (spouses’ sex, age, ethnicity, and socioeconomic characteristics), *enabling* (rurality and insurance type), and *need* characteristics (spouses’ pre-existing MHCs, decedents’ stage at cancer diagnosis, survival, and cancer decedents’ healthcare utilization) and the outcome of MHCs.

#### MHCs

The outcome of interest was a documented diagnosis of mood- or stress-related disorder documented in administrative data any day after day 1 of the death of the partner. Anxiety, depression, and stress-related disorders were identified with International Classification of Disease (ICD) 9 and 10 codes identified by other large, published studies using administrative records that were developed by or verified with physician input (6, 30) (codes are listed in Supplementary Table 1).

#### Predisposing characteristics

Our primary independent variable was binary sex derived from birth records. We also adjusted the models for age (years), spouses’ ethnicity (non-Hispanic White, Hispanic, and non-Hispanic other ethnicity), census-tract level median annual income quartiles ($16,900–$46,604; $46,605–$60,057, $64,058–$74,624, $74,625–$193,958) obtained from the UPDB’s American Community Survey data, and the maximum education level of decedent as recorded on death certificates (less than high school, high school graduate, some college, college graduate, and postgraduate).

#### Enabling characteristics

Rurality was based on the Utah Department of Health definitions of Urban (counties with a population of 100 persons or more per square mile), Rural (7–99 persons per square mile), and Frontier (<7 persons per square mile) (25). Decedents’ primary insurance type (Medicaid, Medicare, Private, and Other) was also assessed.

#### Need characteristics

We assessed spouses’ pre-existing MHCs and decedents’ stage at cancer diagnosis (localized, regional, and distant) and survival (time from diagnosis to death, in years). Inpatient and emergency department visits after cancer patients’ diagnosis were included as a covariate to assess healthcare utilization.

### Analyses

Descriptive statistics were calculated for the sample, and standardized mean differences were used to compare effect sizes between male and female individuals in the sample. The Kaplan–Meier curves and survival analyses assessing the composite outcome of death and MHCs were conducted, followed by cause-specific analyses, accounting for censoring at death. To avoid overfitting, we included spouses’ sex, age, and history of MHCs, and patients’ cancer stage, survival, education, census-tract level income quartile, inpatient stays, and emergency department visits in the models. To account for the higher use of mental healthcare among female individuals, in general, we included the interaction of sex with mental health condition in the models. After applying the inclusion and exclusion criteria, missing data for each variable were assessed. The proportion of missing information for all variables did not exceed 8%. We assumed missing data to be missing at random and used multiple imputation models to address the missing data (31). After identifying variables with missing data, we used the Hmisc and rms packages in R to impute the data using the AREG method to impute the data and aggregate the information across 23 iterations of the imputed data (32–34). All analyses were conducted in R with significance at a *p*-value of <0.05.

## Results

### Characteristics of the sample

**Figure 2:**
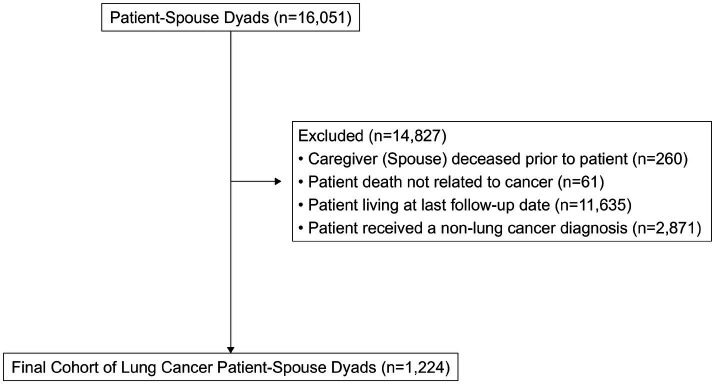
Cohort selection.

A total of *N* = 1,224 spouse-patient dyads (female spouses *n* = 824; male spouses *n* = 400) were identified after excluding lung cancer patients without spousal links (*n* = 31,379) and patients who were living (*n* = 14,824; *n* = 3; Figure 2). Female spouses had a median age of 68 years (interquartile range (IQR) = 61–76), and male spouses had a median age of 70 years (IQR = 64–77; Table 1). The majority of spouses were non-Hispanic White (female *n* = 723, 89%; male *n* = 354, 90%) and lived in urban areas (female 566, *n* = 69%, male = 285, 71%). Almost one in five female and male spouses lived in census tracts with the lowest quartile of annual household income. Greater proportions of male spouses had lost a partner with less than a high school education (19% vs. 17%) and a high school education (37% vs. 33%), while greater proportions of female spouses lost a partner with a post-college education (9.5% vs. 5.5%).

**Table 1:** Differences in characteristics between male and female spouses (*N* = 1,224).

		Spouse sex			
Group	Characteristic	Female	Male	SMD^2^	95% CI^2,3^
		*n* = 824^1^	*n* = 400^1^		
Spouse	Age at Diagnosis (Median, IQR; years)	68 (61, 76)	70 (64, 77)	**−0.16**	−0.28, −0.04
	Ethnicity^4,5^			0.04	−0.08, 0.16
	Non-Hispanic White	723 (89%)	354 (90%)		
	Hispanic	59 (7.3%)	25 (6.3%)		
	Non-Hispanic Other	31 (3.8%)	15 (3.8%)		
	Spouse’s pre-existing mood disorder	54 (6.6%)	21 (5.3%)	0.06	−0.06, 0.17
Patient	Rurality^4^			0.06	−0.06, 0.18
	Urban	566 (69%)	285 (71%)		
	Rural	207 (25%)	95 (24%)		
	Frontier	50 (6.1%)	20 (5.0%)		
	Education^4,6^			**0.17**	0.05, 0.29
	Less than high school	136 (17%)	75 (19%)		
	High School Graduate	274 (33%)	146 (37%)		
	Some College	248 (30%)	114 (29%)		
	College Graduate	84 (10%)	41 (10%)		
	Postgraduate	78 (9.5%)	22 (5.5%)		
	Census-tract level median income (Dollars)			0.09	−0.03, 0.21
	16,900–46,604	154 (19%)	82 (21%)		
	46,605–60,057	270 (33%)	115 (29%)		
	64,058–74,624	231 (28%)	115 (29%)		
	74,625–193,958	169 (21%)	88 (22%)		
	Stage^4,7^			**0.22**	0.10, 0.34
	Distant	532 (67%)	286 (73%)		
	Localized	136 (17%)	38 (9.7%)		
	Regional	128 (16%)	66 (17%)		
	Insurance type^4^			0.11	−0.01, 0.23
	Medicaid	25 (3.1%)	16 (4.1%)		
	Medicare	580 (73%)	268 (68%)		
	Other	13 (1.6%)	8 (2.0%)		
	Private	179 (22%)	103 (26%)		
	Inpatient visits post-diagnosis^4^	3.0 (1.0, 6.0)	3.0 (1.0, 6.0)	0.05	−0.08, 0.17
	Emergency room visits^4^	1.0 (0.0, 3.8)	2.0 (0.0, 4.5)	−0.08	−0.20, 0.04

^1^Median (IQR); *n* (%). ^2^SMD, standardized mean difference; bolded values are significant. A difference of 0.2 indicates a small, 0.5 a medium, and 0.8 a large effect size (34). ^3^CI, confidence interval. ^4^Indicates unknown/missing data, numbers suppressed due to small cell sizes to protect confidentiality. ^5^Due to small cell sizes, ethnicity was combined into three categories. Non-Hispanic and Others included individuals classified as American Indian/Alaska Native, Asian, Black or African American, Native Hawaiian or Pacific Islander, or Multiple Ethnicities and Non-Hispanic. ^6^Decedents’ lifetime maximum level of education was assessed using death certificate data. ^7^Reflects the stage of cancer diagnosis as indicated in Utah Cancer Registry records.

Pre-existing MHCs prior to decedents’ deaths were observed in 6.6% of female (*n* = 64) and 5.3% (*n* = 21) of male spouses, respectively. A greater proportion of cancers diagnosed at distant stages was observed for deceased partners of both female and male spouses (67% and 73%, respectively), and the majority of decedents were insured through Medicare (partners of female spouses *n* = 580, 73%; partners of male spouses *n* = 268, 68%). Deceased partners of female and male partners had a median of 3 inpatient visits and 1–2 emergency department visits after their cancer diagnosis, with a majority of decedents dying at home (62%–64%). Female spouses were significantly younger (standardized mean difference, SMD = −0.16, 95% CI = −0.28–0.04), differed in deceased partners’ maximum education level (SMD = 0.17, 95% CI = 0.05–0.29), and cancer stages (SMD = 0.22, 95% CI = 0.10–0.34), which supported the fact that these differences were not large in magnitude.

### Events (composite outcome of MHCs and death)

**Figure 3:**
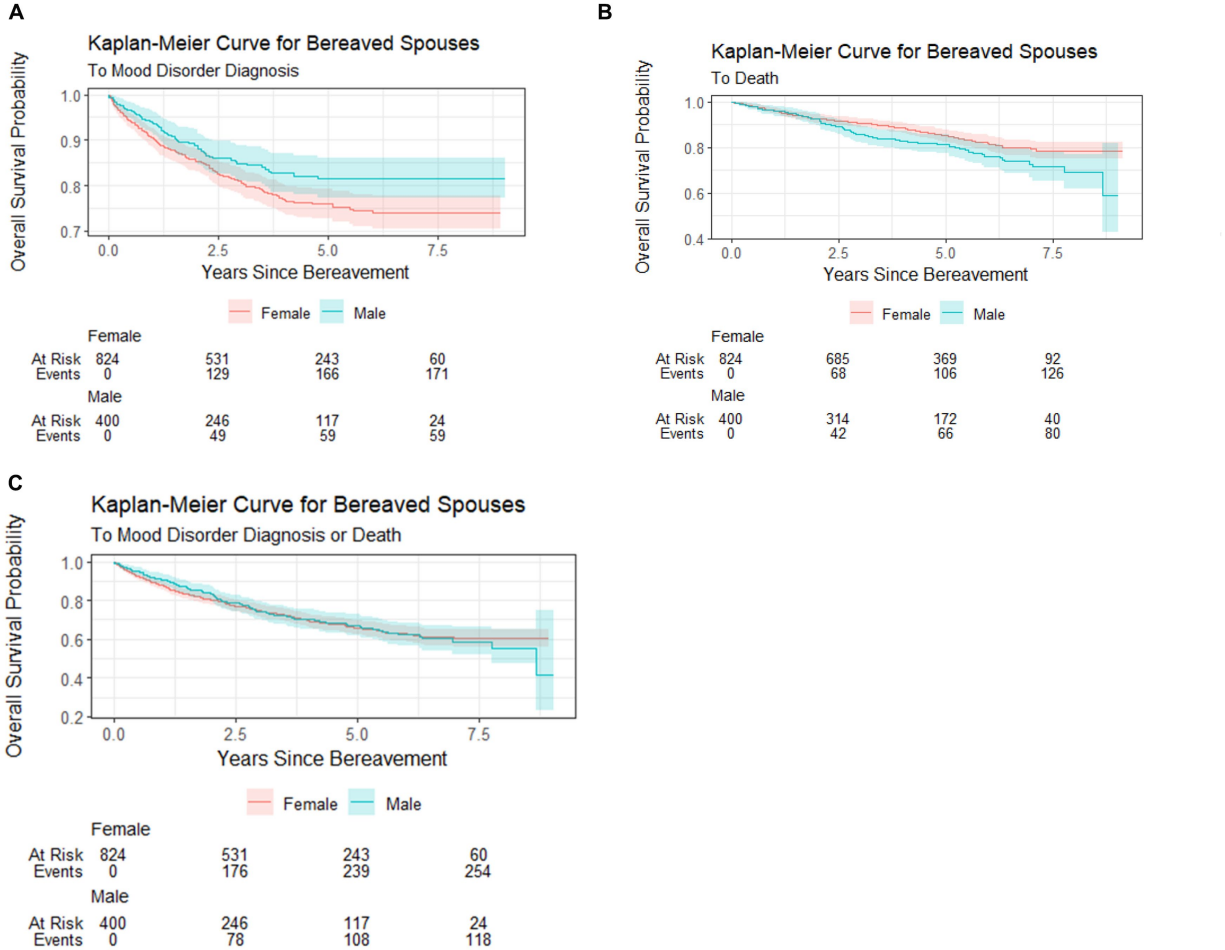
Kaplan–Meier curves for overall probabilities of MHC, death, and MHC + death. **(A)** Probability of MHC by bereaved spouses’ sex. **(B)** Probability of death by bereaved spouses’ sex. **(C)** Probability of MHC + death by bereaved spouses’ sex.

To account for the possibility of death as a competing event to MHCs, i.e., spouses may die prior to presenting to a health facility where MHCs would be documented, we developed a composite outcome of MHC and deaths. A total of 374 events contributed by *N* = 1,224 spouses were documented across the study period. Figure 3 features the Kaplan–Meier curves by sex that describe the estimated overall probability of mood disorder or composite outcome (mood disorder or death) over time (years). The curves suggest that female spouses had a greater probability of mood disorder (Figure 3A), male spouses had a greater probability of death (Figure 3B), and that male and female spouses had a similar probability of any event (mood disorder + death; Figure 3C).

### Cox proportional hazards models

Separate Cox proportional hazards models evaluated differences in time to the composite outcome and mood disorder accounting for censorship due to death (cause-specific model). After adjusting for the interaction between sex and MHCs, neither model showed a difference in the risk of MHCs in bereavement based on sex or decedents’ healthcare utilization.

#### Model for the composite outcome

After adjusting for covariates, the risk of MHC/death was increased for each 10-year interval for surviving spouses. Specifically, at a given point in time, a 10-year increase in spouses’ age at diagnosis was associated with a hazard ratio of 1.37 (95% CI: 1.19, 1.55). Having a diagnosed MHC prior to the cancer decedents’ death was associated with a three times higher risk (aHR: 3.16, 95% CI: 2.21, 4.53) of MHC/death. Compared with decedents whose maximum lifetime education was at the high school level, spouses of decedents with some college-level education (aHR: 0.69, 95% CI = 0.52–0.90) and who were college graduates (aHR: 0.64, 95% CI = 0.41–0.99) had a lower risk of MHCs in bereavement (Table 2).

**Table 2:** Adjusted Cox proportional hazards for the composite outcome (time to MHC or death).

Variables	aHR^1,2^	S.E.	Lower 0.95	Upper 0.95
Male sex (spouse)	0.96	0.11	0.76	1.20
Spousal age at patients’ diagnosis, per 10-year difference	**1.36**	0.07	1.19	1.55
Spouse’s pre-existing MHC	**3.16**	0.18	2.21	4.53
Time from Diagnosis to Death	0.95	0.05	0.85	1.05
Decedents’ maximum education, ref. High school graduate				
College Graduate	**0.64**	0.23	0.41	0.99
Less than high school	1.09	0.14	0.83	1.43
Postgraduate	0.81	0.23	0.52	1.27
Some College	**0.69**	0.14	0.52	0.90
Decedents’ inpatient visits after cancer diagnosis	0.99	0.02	0.96	1.02
Decedents’ emergency department visits after cancer diagnosis	1.01	0.01	0.98	1.03

^1^Models have been adjusted for decedents’ census-tract level income, cancer stage at diagnosis, maximum lifetime education, insurance, and the interaction of sex*MHC (all not significant). ^2^Bolded coefficients indicate statistical significance.

#### Cause-specific model

In the cause-specific analysis, spouses’ age was no longer a significant predictor of the risk of an outcome; however, the decedents’ length of survival was associated with spouses’ risk of MHCs. An additional year of survival was associated with a 15% decrease in the risk of spouses experiencing an MHC in bereavement. As in the previous analysis, having a previously diagnosed MHC was associated with a significantly adjusted hazard ratio of 4.09 (95% CI: 2.70, 6.19). Spouses of decedents with some college-level education had a lower risk of bereavement MHCs (aHR: 0.68, 95% CI = 0.48–0.97) compared with decedents with a high school-level education, after accounting for censorship due to death (Table 3).

**Table 3:** Adjusted Cox proportional hazards for the cause-specific model (time to MHC adjusted for censorship due to death).

Variables	aHR^1,2^	S.E.	Lower 0.95	Upper 0.95
Male sex (spouse)	0.75	0.15	0.56	1.02
Spousal age at patients’ diagnosis, per 10-year difference	0.94	0.08	0.80	1.10
Spouse’s pre-existing MHC	**4.09**	0.21	2.70	6.19
Time from Diagnosis to Death	**0.85**	0.07	0.74	0.99
Decedents’ maximum education, High school graduate				
College Graduate	0.76	0.26	0.46	1.27
Less than high school	1.18	0.18	0.83	1.67
Postgraduate	0.88	0.28	0.51	1.50
Some College	**0.68**	0.18	0.48	0.97
Decedents’ inpatient visits after cancer diagnosis	1.00	0.02	0.97	1.04
Decedents’ emergency department visits after cancer diagnosis	1.01	0.017	0.98	1.05

^1^Models have been adjusted for decedents’ census-tract level income, cancer stage diagnosis, insurance, and the interaction of sex*MHC (all not significant). ^2^Bolded coefficients indicate statistical significance.

## Discussion

Examining the impact of bereavement MHC over time is important for cancer spouses—a population at greater risk of developing psychiatric disorders after the death of their spouse. This was one of the few population-based studies in the US that examined diagnoses of MHCs among lung cancer spouses after the death of a partner.

Bereaved lung cancer spouses have been observed to be particularly at risk compared to non-cancer caregivers and even caregivers of decedents with other cancer types, and lung cancer caregivers’ distress can exceed even that of patients themselves (3). Hu et al.’s analyses of two national registries in Sweden and Denmark observed that spouses of lung cancer had a 45% higher risk of new MHCs compared to spouses of patients with less aggressive cancers (e.g., prostate cancer spouses HR = 1.05, any cancer HR = 1.14) after the diagnosis of cancer in their partners (6). However, there is limited epidemiologic research comparing differences in bereavement MHCs by cancer types in the US, except for a few. A 2021 study by Hess et al. using IBM MarketScan Administrative Data in the US observed that lung cancer caregivers had the highest proportions of new MHC-related diagnoses (18.9%) in the first year after a cancer patient’s diagnosis compared with gastric (17.8%), colorectal (14.7%), sarcoma (14.3%), and breast cancer (10.1%) caregivers (7). Taken together, these findings support that lung cancer spouses may be a particularly at-risk caregiving population; however, future research comparing across cancer types is needed to verify these outcomes among spouses of other cancer patients in the US population.

While we anticipated that the female sex of the surviving spouse would be associated with greater MHCs, our cause-specific analysis did not find a statistically significant difference after accounting for earlier mortality in male spouses. This finding may be due to the lower reporting and help-seeking behaviors of male individuals for MHCs in general (35). Our previous studies examining prescriptions of antidepressants and anxiolytics in families of decedents in the last year of life observed higher proportions of antidepressant prescriptions among wives compared with husbands (36), supporting the possibility that female individuals seek more care for MHCs. However, gender differences in the timing of symptoms may influence these findings. Other registry-based studies have found that male individuals exhibit prolonged grief symptoms more acutely, while female individuals have a more protracted trajectory of symptoms (20). Given our observation of earlier mortality in male individuals, there is a possibility that more acute and severe mental health symptoms associated with earlier mortality in male individuals may have affected these findings. While outside the scope of our study, future studies comparing outcomes with a non-bereaved matched cohort are recommended to better distinguish the gender effect of bereavement on mental health and mortality.

Losing a spouse is among the most difficult life events for adults, and 10–20% of bereaved spouses will develop severe or prolonged grief responses (37). Our findings support the finding that having a previously diagnosed mood or stress-related disorder was associated with over four times higher risk of having an MHC, which is in line with findings from other survey-based epidemiologic studies of complicated grief (38). Future studies should also examine serious mental health diagnoses such as schizophrenia, bipolar, major depressive disorders (39), or co-occurring substance or alcohol use disorders (6, 40, 41) to better identify the most at-risk individuals who may benefit from targeted support in bereavement.

We focused on MHCs that were documented in administrative data, which may reflect a sample with more severe MHC who were diagnosed or treated in healthcare settings (42). It is important to note that these data may neither capture mental health outcomes for individuals with subclinical but meaningful levels of symptoms nor cases if individuals do not seek health care for their MHCs. A study of 168 bereaved cancer caregivers of lung and gastrointestinal cancer patients found that 30.4% of caregivers self-reported clinical levels of depressive symptoms, while 43.4% reported clinically significant levels of anxiety after the death of their loved one (43). Thus, it is possible that the prevalence of MHCs may be higher in the bereaved lung cancer spouse population than these findings observed.

The greater risk of MHC in this caregiver population may be due to the unique stresses of lung cancer caregiving (4, 10, 11). While we hypothesized that greater overall healthcare utilization in deceased patients would be associated with greater bereavement MHC, this association was not supported in our findings. A possibility may be that we assessed the number of inpatient and emergency department visits beginning after patients’ cancer diagnoses, which may fail to capture the more stressful contexts of high-intensity care that cluster toward the end of life (44). In addition, dyadic effects may also exist, as poorer bereavement mental health in caregivers has been associated with perceived distress in their loved one prior to death (43). These factors associated with the end-of-life care context would be important to examine in future analyses.

Population studies observed that bereaved cancer spouses have an increased risk of psychiatric disorders if their partners had advanced cancers and cancers with poorer prognoses (6). Our findings add that longer survival among cancer decedents was associated with a lower risk of MHCs in bereavement. A possibility is that longer survival may facilitate greater emotional adjustment in spouses of cancer patients, particularly when death is expected (45). This finding is in contrast with our previous analysis of the hospice patient population that observed that longer hospice duration was positively associated with mortality risk for bereaved husbands but not wives in the general population (46). These findings collectively suggest that gender and trajectories of decline may play a modifying role in the end-of-life cancer caregiving stress experience.

Although we adjusted for multiple factors, our findings were unable to account for psychosocial factors or cultural influences on bereavement that can serve a protective or risk role. For example, perceived interpersonal support is protective for psychological wellbeing and health-related quality of life among cancer caregivers (47, 48). Additionally, caregiving and subsequent bereavement may be perceived as normative and a fulfillment of a spousal responsibility, influenced by certain cultural or ethnic backgrounds (49). Conversely, some cultures and communities may hold beliefs that discourage seeking bereavement support (50) or mental health services more broadly (51). Future research integrating self-reported or qualitative methods and exploring bereavement mental health outcomes in more racially and ethnically diverse samples may be helpful in distinguishing the contribution of psychosocial and cultural influences.

Finally, while our study did not have access to individual-level household income, our findings supported that socioeconomic status may be protective of spousal bereavement MHC. While indirect, this finding supports the implications that dyadic factors may continue to influence surviving members of dyads after the death of a partner. The higher level of educational attainment may be associated with greater income or wealth for the surviving partner, which should be examined in future studies using individual-level measures of socioeconomic status. Examining the socioeconomic influences associated with bereavement is important as financial hardship in surviving spouses has been associated with an increased risk of suicidal ideation (38).

### Limitations

While the strengths of this study were a population sample and the use of objective data, limitations include the retrospective nature of the study and the potential for detection bias. Individuals who do not have healthcare encounters in inpatient, ambulatory surgery, or emergency department settings or do not seek care for MHCs are not represented in these data. Studies using self-reported data may observe different findings. Future studies should evaluate MHCs using other measures, including self-reported measures. Due to the smaller sample and rarer occurrences of MHC, we limited the examination of additional predictors to prevent overfitting of the data. Thus, there is a possibility of potentially unmeasured confounders, such as access to social support, cultural factors, and the end-of-life caregiving context. Additionally, data from this study were specific to a single state, which poses implications for generalizability. Nevertheless, population data allow us to minimize selection bias compared with smaller, prospective samples, which is a strength of this study.

## Conclusion

Bereavement is a major life stressor that can contribute to mental health morbidity and is one of the major stressors associated with serious illness caregiving. Building population-level evidence for the pervasive impacts of cancer caregiving is important to guide care delivery across the cancer continuum and beyond. Future research should investigate these outcomes in a more nationally representative sample and investigate other mental health outcomes that may be relevant to difficult loss. Nevertheless, our findings hold implications of greater mental health assessment and support for bereaved partners that may be at risk for greater mental health challenges after the loss of a spouse to lung cancer, such as lung cancer spouses that have pre-existing MHCs.

## Data Availability

The data analyzed in this study is subject to the following licenses/restrictions: use of the data held by the Utah Population Database (UPDB) and its data contributors are regulated. Under an Executive Order, the Utah Resource for Genetic and Epidemiologic Research (RGE) is responsible for regulatory oversight of all projects using data from the UPDB. Access to datasets from the UPDB (including those constructed for this application) is governed and approved by the RGE committee, which includes representatives of the data providers whose data are included in UPDB. An RGE protocol is ancillary to an Institutional Review Board (IRB) protocol, which may be held external to the University of Utah. Data may only be transferred from the University of Utah to an external institution with RGE approval and a fully executed data transfer agreement. Requests to access these datasets should be directed to Nicola Camp, Nicola.camp@hci.utah.edu.
