# Supplementary material for: Risk of mental health conditions in bereavement: a population-based analysis of lung cancer spouses
Source: Front Public Health. 2025 May 12;13:1539180. doi: 10.3389/fpubh.2025.1539180 (PMC12104072; doi:10.3389/fpubh.2025.1539180)
Supplement: Supplementary file 1 [file Table_1.docx]

**Supplementary Table 1. International Classification of Disease (ICD) Codes**

| **Psychiatric disorder** | **ICD-9 codes** | **ICD-10 codes** | **Data source** |
| --- | --- | --- | --- |
| Depressive disorder^5^ | 296.20-296.25 296.30-296.35  296.50-296.55  300.4x, 309.0x 309.28, 311.xx | F32.9  F33.0-F33.3 F33.40, F33.41 F33.9, F32A F31.30-F31.35 F34.1, F43.2x  F32.9 | - Utah Department of Health and Human Services Health Facility Records (Inpatient, Ambulatory Surgery, and Emergency Department) - All-Payer Claims Database |
| Anxiety disorder^5^ | 300.00-300.02 300.21-300.23  300.29, 300.3x 308.3x, 309.24, 309.81 | F41.0-41.1, F41.9  F40.01-40.02, F40.10  40.8, F42  F43.22-F43.23  F43.1, F43.12 | - Utah Department of Health and Human Services Health Facility Records (Inpatient, Ambulatory Surgery, and Emergency Department)   All-Payer Claims Database |
| Stress-related disorders^24^ | 308-309 | F43 | - Utah Department of Health and Human Services Health Facility Records (Inpatient, Ambulatory Surgery, and Emergency Department)   All-Payer Claims Database |
